# Metacarpophalangeal joint arthritis in IgA vasculitits

**DOI:** 10.1093/omcr/omae157

**Published:** 2024-12-28

**Authors:** Yamato Hanawa, Hiroyuki Namba

**Affiliations:** Department of Pediatrics, Jikei University School of Medicine, Tokyo, Japan; Department of Pediatrics, Jikei University Kashiwa Hospital, Chiba, Japan; Department of Pediatrics, Jikei University School of Medicine, Tokyo, Japan; Department of Pediatrics, Jikei University Kashiwa Hospital, Chiba, Japan

**Keywords:** Immunology, Rheumatology

A previously healthy 4-year-old boy was referred to our hospital, the physical examination revealed warmth, pain, and swelling in bilateral ankle joint and metacarpophalangeal joints 2–4 of right hand (Fig. 1), as well as palpable purpura in the lower bilateral extremities (Fig. 2). He also complained of diffuse abdominal pain and hematochezia. At admission, the laboratory test showed the elevation of D-dimer and normal platelet. He showed no hematuria and renal dysfunction. Based on the EULAR/PRINTO/PRES criteria, we diagnosed him as immunoglobulin A vasculitis (IgAV) and administered intravenous methylprednisolone, and clinical manifestations improved dramatically. The intravenous methylprednisolone was tapered, and he was discharged.

**Figure 1:**
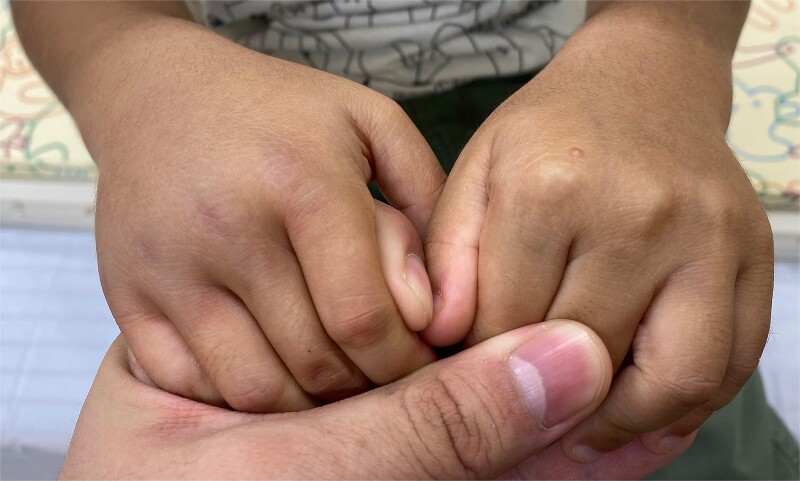
Right metacarpophalangeal joint swelling.

**Figure 2:**
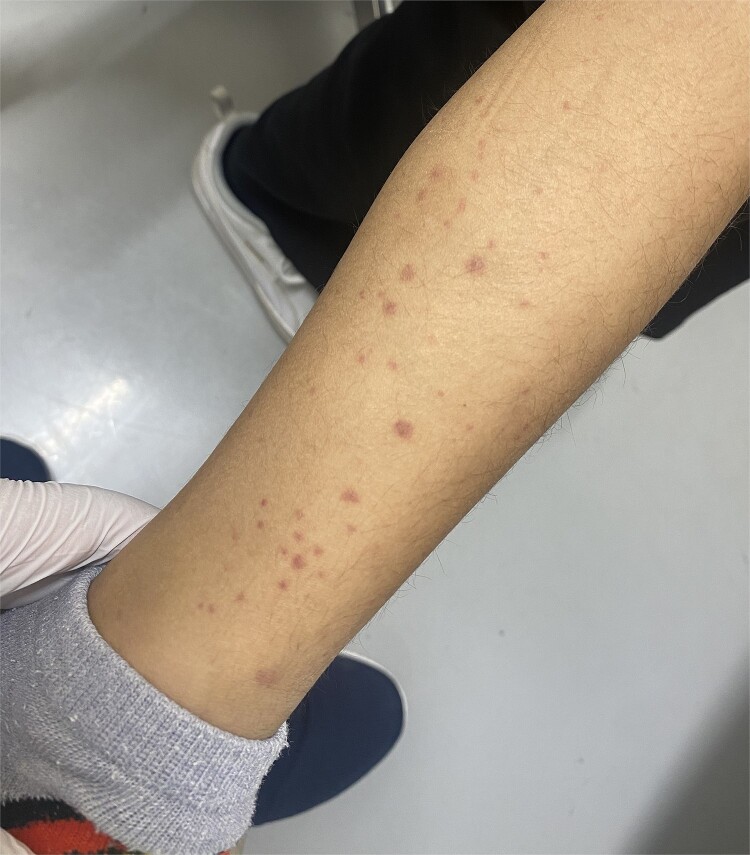
Palpable purpura in the lower limb.

IgAV, formerly known as Henoch-Schönlein purpura, is an acute small vessel vasculitis. The most common age range is 5–10 years old, and males are affected more often than females [1]. Typical clinical symptoms include palpable purpura, arthritis/arthralgia, gastrointestinal involvement, and renal involvement. Joint involvement is the second most common feature, occurring in roughly 75% of patients and most often affecting large joints of the lower extremities such as knee, ankle and hip [2]. The joints of the upper extremities are involved in a few patients. There is usually prominent periarticular swelling, tenderness and pain; erythema and joint effusion are rare. Arthritis is non-deforming and heals without chronic damage within a few weeks [3, 4]. It is important to remember that arthritis may precede the onset of the purpura by up to a week in 15%–25% of patients [2].

Upper joint arthritis in IgAV is comparatively rare, but it can contribute to the diagnosis of IgAV [2]. Based on the experience of this case, it was demonstrated that when observing arthritis of the upper limbs, we should consider IgA vasculitis and be attentive to the appearance of purpura.

## Consent

The parent of this patient has given written informed consent for the publication of this manuscript and images.

## Gurantor

Yamato Hanawa.
